# Paths to research-driven decision making in the realms of environment and water

**DOI:** 10.1016/j.techsoc.2022.101994

**Published:** 2022-08

**Authors:** Savannah Cooley, Amber Jenkins, Blake Schaeffer, Kat J. Bormann, Adel Abdallah, Forrest Melton, Stephanie Granger, Indrani Graczyk

**Affiliations:** a NASA Western Water Applications Office, Applied Sciences Program, United States; b Jet Propulsion Laboratory, California Institute of Technology, United States; c U.S. Environmental Protection Agency, United States; d Airborne Snow Observatories, Inc, United States; e Western States Water Council, United States; f NASA Ames Research Center Cooperative for Research in Earth Science and Technology, United States

## Abstract

Now more than ever it is critical for researchers and decision makers to work together to improve how we manage and preserve the planet’s natural resources. Water managers in the western U.S., as in many regions of the world, are facing unprecedented challenges including increasing water demands and diminishing or unpredictable supplies. The transfer of knowledge (KT) and technology (TT) between researchers and entities that manage natural resources can help address these issues. However, numerous barriers impede the advancement of such transfer, particularly between organizations that do not operate in a profit-oriented context and for which best practices for university-industry collaborative engagement may not be sufficient. Frameworks designed around environmental KT – such as the recently-developed Research-Integration-Utilization (RIU) model – can be leveraged to address these barriers. Here, we examine two examples in which NASA Earth science satellite data and remote-sensing technology are used to improve the management of water availability and quality. Despite differences in scope and outcomes, both of these case studies adopt KT and TT best practices and can be further understood through the lens of the RIU model. We show how these insights could be adopted by NASA through a conceptual framework that charts individual- and organizational-level integration milestones alongside technical milestones. Environmental organizations can learn from this approach and adapt it to fit their own institutional needs, integrating KT/TT models and best practices while recognizing and leveraging existing institutional logics that suit their organization’s unique history, technical capability and priorities.

## 1. Introduction

Transferring environmental research to decision makers can help address multi-faceted natural-resource management challenges that are increasingly exacerbated by climate change [1,2]. Natural resources such as water may be considered a common pool resource, where communities operate within complex social and legal frameworks that enable the use of finite resources [3–5]. Human-driven climate change compounds these common pool resource challenges [6]. Water managers in the western U.S, for instance, must grapple with increases in the frequency and intensity of droughts and floods [7]. These hydrologic extremes complicate the already challenging task of deciding how best to allocate water resources where demand outstrips supply [8]. Monitoring and forecasting the impacts of natural-resource use can potentially reduce over-consumption and provide decision makers with valuable feedback on management decisions (e.g., [9]. Earth-science research and technology has an opportunity to help advance these capabilities.

Despite the promise of potential for KT/TT between Earth-science research and environmental decision-making, many longstanding barriers remain. The mechanisms by which academic knowledge is transferred across organizational boundaries in university-industry collaborations are well documented and many success stories exist [10]. There is a need for a similar set of successful pathways for transferring research to improve the management of natural resources. In 2003, the U.S. National Research Council published a report on accelerating KT/TT within and across the National Aeronautics and Space Administration (NASA), the National Oceanic and Atmospheric Administration (NOAA) and Department of Defense (DoD) [11]. It found that the processes by which Earth-science knowledge is transferred between these agencies are largely ad hoc, lacking the structure needed to ensure KT/TT is efficient and effective. Several key barriers were identified. For example, the potential value of the new information or technology is often neither clearly defined nor articulated for the new setting in which it could be used, creating a lack of incentives to alter existing approaches. Other hurdles include cultural differences between the environmental research and decision-maker communities, organizational and communication issues, inadequate scientific or technological capability, and lack of long-range financial resources coupled with technical planning. Today, nearly two decades later, many of these barriers to transferring Earth-science expertise and local knowledge still exist, as identified in numerous recent studies [12–17].

To overcome these barriers and address urgent natural-resource issues, organizations conducting environmental research can benefit from adopting well-established KT/TT insights. These include recognizing the critical role of building relationships and trust between individuals [18–20], cultivating effective communication [21,22] and fostering champion behavior [23–25].

However, recent work highlights that solely focusing on individual-level best practices fails to address the importance of institutional processes and differing motivations of researchers versus decision makers in environmental KT [12]. Generally, scientists in academia orient around the incentive system of open science and publications, while those in industry face the imperative to produce results with commercial benefits. Despite underlying diverging incentives, university-industry collaboration is widely and successfully practiced [10]. We propose a similar opportunity exists for pervasive KT/TT among environmental research and operational entities, and it is yet to be realized. To catalyze this possibility, environmental research and stewardship organizations can learn from best practices within the university-industry KT/TT field, while also recognizing that a parallel but unique contrast in incentive systems must be directly confronted [12].

Natural-resource management often consists of common pool resource instances where regulation is important for optimal resource use and there may be limited or no explicit market for the resource [4]. As a result, environmental management may not offer viable commercial opportunities and is instead often facilitated through intervention from central governments, non-profits, or through traditional, self-organized group-property regimes [3]. While such entities may not be profit-driven, their motivations may differ from those of scientists, being typically oriented around power and feasibility [25] as well as long-term, sustainable management of the resource [5]. The Research–Integration–Utilization (RIU) model [12] reconciles the differences in incentives via “integration”, which is the collaborative, bi-directional selection of research results that are relevant in practice. In emphasizing integration, the RIU model pushes back on the notion stated in many KT/TT guidelines that the crucial part of KT involves “communication” and other skills that can be cultivated at the individual level (e.g., [26]. Instead, Bocher & Krott [12] suggest that a much more important aspect for environmental KT/TT is the processes that can connect science-based information to the resources of political and practical actors (i.e., “integration”). If the science-based information meets a practical need, then policy and decision-making entities will use their resources to promote the use of said knowledge in their specific application. If, on the contrary, science-based information is not useful to the actors and their interests, then rarely do polished communication or trusting relationships alone lead to environmental KT/TT.

Here, we illustrate the need for additional focus on such integration activities and organizational support for effective environmental KT/TT. We use two water-related case studies to do this: the Airborne Snow Observatory (ASO [27]; and the Cyanobacteria Assessment Network (CyAN; [28]. These two case studies were highlighted at a recent multi-agency workshop [14] led by NASA and the Western States Water Council (WSWC), which was attended by water leaders from NASA, WSWC, federal (USGS, NOAA, USBR, EPA) and state agency partners (representing CA, WY, OR and NE). The first case study charts ASO’s path from a NASA research project to a private company. Although ASO transitioned to a company, its business model depended on government support, causing deviations in incentives from traditional free-market contexts. The second case study – CyAN – details KT/TT across several U.S. government research and management programs that support water-quality monitoring under the U.S. Clean Water Act and Safe Drinking Water Acts. Both case studies excel at RIU. They fill critical water-management information gaps with sound science, while at the same time cultivating strong relationships with champions (“allies”) who use their power to persuade other actors to apply the science to their decision making.

We advance the field of environmental KT/TT by showing how NASA’s Earth Science Division (ESD) might translate these insights and best practices into institutional change. This is done by expanding on a NASA-specific conceptual framework of KT/TT that builds on language, mainly on best practices associated with “integration” and “utilization” since NASA already has a strong foundation for planning and conducting rigorous scientific research. Here, the rigorous scientific research is equivalent to the RIU model “research” component. By aligning our framework with existing NASA institutional history and culture, we can boost NASA’s acceptance of the organizational change needed [29]. The structure of our framework folds in both internal and external institutional “logics” (i.e., language, practices and culture) for KT/TT. We conclude with a vision for how or KT or TT within their own institutions.

### 1.1. Case study #1: the Airborne Snow Observatory (ASO)

**Fig. 1.**
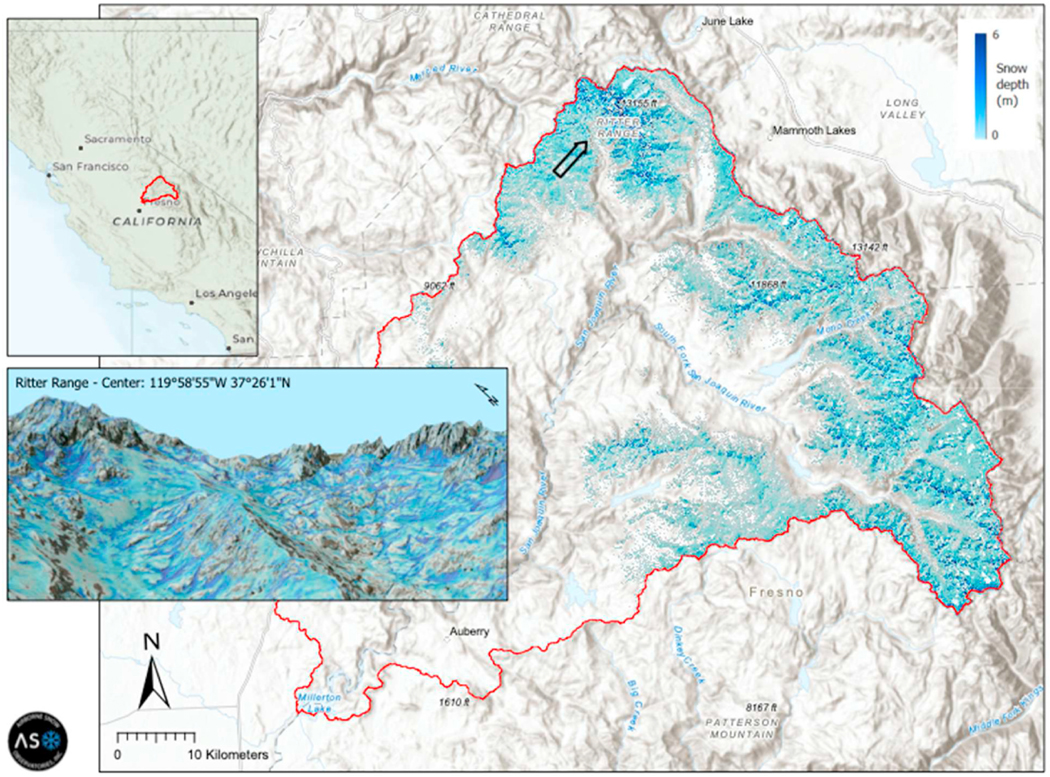
Measurements of snow depth collected on May 4–5, 2020 for the San Joaquin River Basin upstream of Millerton Lake. ASO measured the entire snowpack within the 4500 km^2^ watershed area and recorded snow depth at a spatial resolution of 3 × 3 m and SWE at a resolution of 50 × 50 m (not shown) within 72 h of the survey. The 3D inset highlights the spatial complexity in snow depth in the Ritter Range vicinity, where darker blue reflects deeper snowpack. (For interpretation of the references to colour in this figure legend, the reader is referred to the Web version of this article.)

Some environmental research efforts addressing natural-resource stewardship fit the well-documented path of KT/TT from a research project into a commercial entity. ASO is a case in point. It started in 2012 as a collaborative effort between the California Department of Water Resources (CADWR) and NASA’s Jet Propulsion Laboratory (JPL, a Federally Funded Research and Development Center managed by the California Institute of Technology (Caltech) on behalf of NASA). ASO estimates the amount and distribution of water contained within mountainous snowpack, otherwise known as snow water equivalent (SWE). The mission uses a piloted aircraft to fly a scanning lidar and imaging spectrometer over snow-covered mountains to map SWE at 50-m spatial resolution over watersheds [27]. SWE is derived from the measured snow depth (Fig. 1) combined with a model that uses snow albedo (a measure of its reflectivity) as input. More accurate estimates of SWE help CADWR to model runoff in streams and plan timely state responses to floods or droughts. ASO offers the world’s first accurate measurements of mountain snowpack at regional scales, filling a large void in the snow research community [27].

The commercialization of ASO arose in large part because the program addressed an urgent and well-defined stakeholder need [12, 30–32]. It aligned with established best practice from university-industry entrepreneurship endeavors and more recent environmental KT/TT studies (i.e., “utilization” best practices in RIU model). Water managers in snowmelt-dependent watersheds face unprecedented challenges – more frequent and intense water-cycle extremes and fluctuations in the timing of snowpack melting [7]. The issues posed by climate change, combined with the shortcomings of existing forecasting techniques and a data paucity, called for new information and new solutions. The availability of ASO’s more precise snowpack data fundamentally changed how snowmelt runoff is forecasted and how subsequent reservoir management decisions are made [8].

Another KT/TT principle that proved central to ASO was the significance of champion behavior and allies [12,23,24,33]. Over a decade or more, water managers at regional and state levels and researchers leading ASO championed the advancement of the technology and its uptake. ASO researchers recognized from the onset of their collaboration that the specialized local knowledge and experience of the water management partners was crucial to success. The water management partners helped ASO researchers to understand the physical dynamics of individual watersheds, the reliability of in-situ data for product evaluation, and the timing of when SWE information is most valuable. Cultivating these partners was critical to the effectiveness and impact of the airborne data collection campaigns. ASO strengthened relationships with water managers in parallel with ensuring that the technical requirements were met. In 2020 a consortium of water-partner agencies sought augmented funding to support the acquisition of ASO data through the California state legislature. The initial request for funding to support the ASO Program was approved by the legislature but later vetoed. This veto was not issued from lack of support for the ASO snow surveys, but instead due to concerns with the financial-support mechanisms. The failure to secure funding from CADWR and state legislature ultimately led to other partners joining the conversation and working together to find alternative funding mechanisms. Furthermore, the formation of Airborne Snow Observatories, Inc. – the corporation that runs the ASO program – would not have been possible without the enthusiasm and dedication of the NASA lead scientist to develop a business model, source financial investment and transition to the private sector.

Organizational and institutional arrangements also facilitated the underlying KT/TT for ASO. Again, a parallel between ASO and traditional university commercialization pathways emerges. In both cases, institutional mechanisms play an important role in facilitating the processes by which technology is transferred across organizational boundaries into commercial settings [29,34–36]. For ASO, the primary institutional support mechanisms included the legal, administrative and planning support provided several years before commercialization by NASA’s Commercialization Office, NASA’s Applied Sciences Program and the Caltech Office of Technology Transfer at JPL.

ASO transitioned to a private company and in many ways mirrors university commercialization processes (i.e., intellectual-property creation and academic entrepreneurship). However, its business model depends on government support rather than free-market mechanisms, meaning deviation from traditional free-market KT/TT models. ASO’s main revenue source is government funding at state and federal levels. Even with a dedicated team, strong partnerships, and ongoing unanimous support from the state legislature, ASO’s long-term funding via the legislature has not yet been secured. After several years of ad-hoc funding streams from state and water-district-level entities, the U.S. Bureau of Reclamation announced it would provide $2.5 million to fund research and technology to improve SWE estimation and runoff forecasts. A large portion of this is dedicated to continuing and expanding ASO snow surveys. However, long-term revenue sources for ASO still remain unidentified. ASO’s story illustrates the magnitude and breadth of challenges associated with the financial planning needed to support new, public-benefit technology over the long-term, as well as the gaps in institutional support needed. The complexities involved in developing this unconventional business model and securing funding – a major part of ASO’s commercialization process – are not reflected in traditional KT/TT models or existing NASA support mechanisms.

## 2. Case study #2: the Cyanobacteria Assessment Network (CyAN)

The Cyanobacteria Assessment Network [28] delivers environmental knowledge and technology transfer that falls outside university-industry collaboration or most of commercialization trajectories. Consequently, certain traditional KT/TT principles still apply but often must be supplemented or re-evaluated entirely in order to be relevant to this type of KT/TT.

**Fig. 2.**
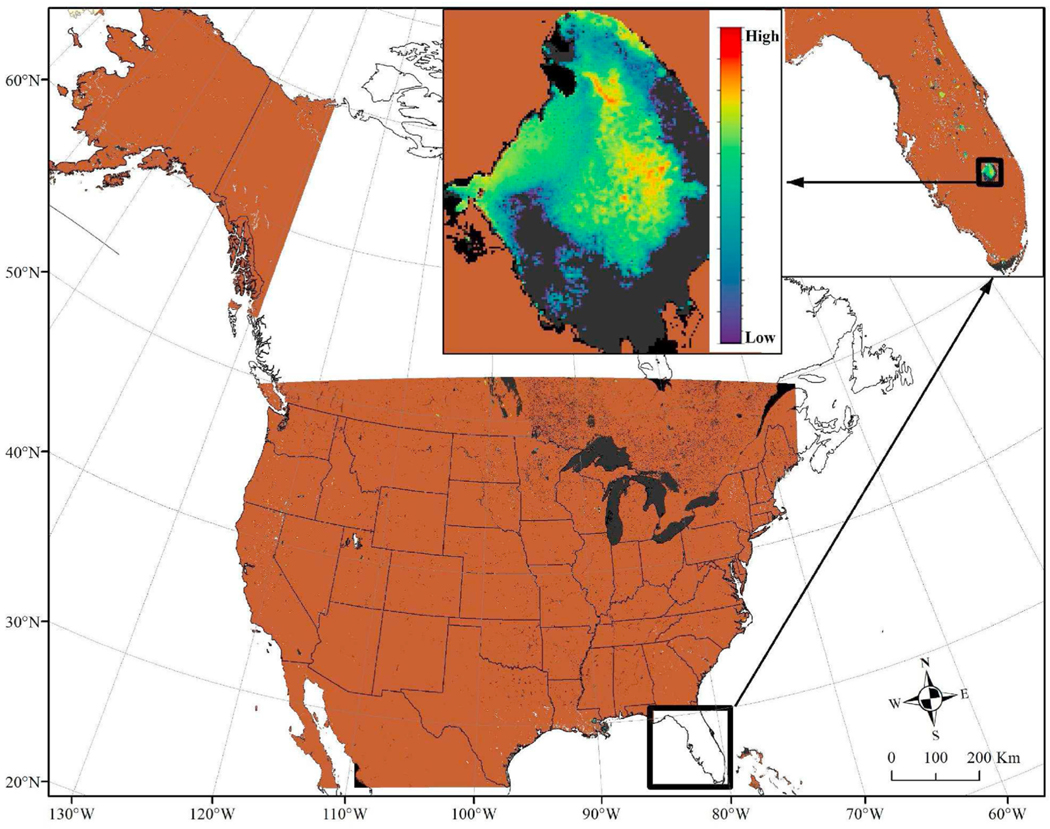
Conceptual representation of the (CyAN) Sentinel-3 satellite coverage for early detection and monitoring of cyanobacterial events across the U.S. The spatial resolution of the data is 300 × 300 m pixels. Operational delivery of cyanobacteria data is available for daily and weekly composites as demonstrated here for the 7-day weekly composite June 23 to June 27, 2020. The figure shows a zoom in of Lake Okeechobee in Florida; blue and green represent low concentrations of cyanobacteria biomass while warm yellow and orange represent higher concentrations. (For interpretation of the references to colour in this figure legend, the reader is referred to the Web version of this article.)

CyAN is a multi-agency project involving the U.S. Environmental Protection Agency (EPA), NASA, NOAA, and the U.S. Geological Survey (USGS) that works with federal, state, tribal and local partners to monitor cyanobacterial blooms using satellite data (Fig. 2). Cyanobacterial blooms may cause harm to human, animal, or aquatic ecosystem health. Previously, few management decisions related to water quality used satellite information because data dissemination to water managers was limited to either photographs or data products that require specialized training to process and interpret [37]. The main satellite-derived information product is a cyanobacteria index algorithm, which separates cyanobacteria from other blooms using the spectral signatures that are sensitive to the presence of phycocyanin [38].

Although not on a path to commercialization, CyAN, as in the case of ASO, addresses an urgent and well-defined resource-management need. The societal benefit that CyAN provides is large, but direct financial motivations for adoption may be more finite. CyAN information is used as a pre-screening tool to direct limited resources to confirm cyanobacteria events and help with a variety of water quality management decisions. The CyAN data quantifies the temporal frequency [39,40], spatial extent [41], magnitude [42], and occurrence [43] of cyanobacteria events across over 2000 U S. lakes and reservoirs. A recent study, which characterized the socioeconomic benefits of CyAN satellite data that were used to manage a 2017 cyanobacterial bloom event, found the benefits to be valued at $370,000 for a single advisory in Utah Lake [44]. Furthermore, the potential avoided costs of using a CyAN-equivalent service for chlorophyll-a monitoring were estimated to be $5.7 million per year [45]. These economic benefits are distributed across society rather than being directed to the agencies responsible for ensuring water quality. Thus, the direct incentives for adoption of CyAN information may be limited to the ecosystem services it supports.

Due to the finite financial incentives, the cultivation of trusting relationships and champion behavior (i.e., “allies” as defined by the RIU model) was perhaps even more important than what has already been shown in traditional university-industry KT/TT [18–20]. In most policy and resource-management organizations, reporting requirements calling for the ongoing integration of new information into the decision process do not exist. This is the case for monitoring cyanobacteria blooms, which CyAN achieves. And even if the need for improved information for decision making is recognized, environmental management organizations typically identify the costs related to data, personnel and resources to use the new information as a major concern [37]. These concerns are especially acute when the adoption of a new technology has no additional funding secured and would require cutbacks in existing programs. Therefore, trust building in these contexts becomes particularly important for the “utilization” step in environmental KT/TT.

Restrictions experienced during a pandemic or any other globally disruptive event may accelerate consideration of new technologies such as satellites. During the COVID-19 pandemic, anecdotal evidence suggested that field teams were hindered in monitoring efforts due to social distancing requirements and travel restrictions. These field teams were assisted by CyAN satellite imagery in the presence of these restrictions.

From the outset, CyAN researchers and collaborators emphasized the importance of trust and relationship building for achieving utilization. Trust was initiated through existing researcher-to-partner relationships, then expanded toward new partners. New partners typically didn’t personally know the CyAN scientist, but derived some amount of trust from the propagation chain [46,47]. Key success factors were the willingness of CyAN researchers to accept input along the way, spend time answering the partners’ questions, and commitment to the entire KT/TT process, rather than only offering the minimum involvement needed to produce a peer-reviewed publication. Rather than solely focusing on the scientific aspects of the project, some CyAN researchers committed 10–20% of their time to relationship building, development of training materials, and answering partners’ technical and general queries. The CyAN researchers also removed most technical language from informal and formal communications to reduce barriers to understanding and avoid exclusionary practices [48]. CyAN scientists openly recognized that they needed to learn from operational partners to develop optimal solutions for cyanobacteria management [37]. For instance, additional quality assurance flags for errors such as mixed land and water pixels or snow and ice that could only be verified by local users experiencing those conditions. Ground-based validation data came primarily from federal and state collaborators [49,50] a categorical approach using trophic state categories [51], and quantitative approach using cyanobacteria [39] and chlorophyll concentrations [51]. Operational partners also provided beta testing and feedback on all the data products and software uses [52]. Qualitative applied measures of trust due to the co-development framework were captured in collaborator comments regarding CyAN’s efforts in a NASA impact report [53].

Support for CyAN at the institutional level also played an important role in its success. Such support included coverage of the mobile and web-based application hosting costs [52]; software maintenance support; mutually-beneficial hosting of Landsat surface temperature data; funding of open-access publication charges to democratize the access of the peer reviewed science publications; use of well-established public webinar platforms to reach potential new users and recruit new allies; access to a previously-established network of harmful algal bloom managers and local water managers; and technical assistance (e.g. through the Nutrient Scientific Technical Exchange Partnership & Support program). The diversity of institutionally supported outreach platforms provided scientific researchers a broader audience than was originally planned. Subsequent question and answer meetings were typically scheduled with smaller groups to address specific application needs and requirement. As a result of this support, satellite remote-sensing-based methods for cyanobacterial monitoring were incorporated by stakeholders far beyond the immediate CyAN collaboration. Satellite remote sensing approaches to monitoring water quality are now included in recommendations by the World Health Organization (Welker et al., 2021) and Interstate Technology Regulatory Council [54].

## 3. A NASA KT/TT framework

Using insights from the case studies presented in the context of the KT/TT field, we now work to improve KT/TT at NASA by developing a revised framework. To do this, we leverage existing institutional KT/TT knowledge and efforts previously established within the agency because the added cultural buy-in increases chances of uptake [29]. We build on NASA’s Applied Sciences Program “Application Readiness Level” (ARL) scale (Figure S1; [55], a framework constructed with nine steps to assess the maturity of projects oriented towards applied science and KT/TT. The ARL scale, which is an analog of the longstanding NASA Technical Readiness Level (TRL) scale (Figure S2; [56,57]), describes the research steps and technical milestones in three phases that closely resemble the standard stage-gate process [58]. ARLs 1–3 generally cover discovery and feasibility, ARLs 4–6 address development, testing and validation, and ARLs 7–9 focus on integration of the “application” into a decision-making activity. The nine ARLs are:

1. identification of basic research application (baseline ideas);
2. development of application concept (invention);
3. proof of concept (viability established);
4. initial integration and verification (prototype);
5. validation in relevant environment (potential determined);
6. demonstration in relevant environment (potential demonstrated);
7. use of application prototype in partner’s decision making (functionality demonstrated);
8. completion of application (functionality proven);
9. operational deployment and use in decision making (sustained use).

NASA’s original ARL scale can benefit from including individual and organizational-level relationship-building, two-way knowledge exchange or co-development processes associated with effective integration and utilization for environmental KT/TT [1,11,12,31,59]. The ARL scale can also be improved by recognizing that the nine levels are neither fully discrete nor sequential. The modified framework presented here – referred to as the “R2O–O2R framework” – addresses these gaps.

Here, we adopt the term “research to operations” (R2O) in place of KT or TT to align with language commonly used by federal agencies responsible for satellite missions [11]. The term “operations to research” (O2R) refers to when operational (i.e., decision-maker) needs motivate and inform research efforts, thus emphasizing a two-way knowledge exchange grounded in the theory and practice of co-developed integration processes. Many definitions and applications of co-development exist across fields ranging from design research [60,61], international development [16,62], and immigration policy [63]. These definitions coalesce around the cultivation of relationships between two or more stakeholders with the aim of achieving a set of common, mutually-valued and practical objectives. Defining and reaching these objectives (i.e., successful “integration”) requires some degree of shared power, decision making and ownership of outcomes, where all participating actors agree to invest resources – including knowledge – toward the collaborative effort.

**Fig. 3.**
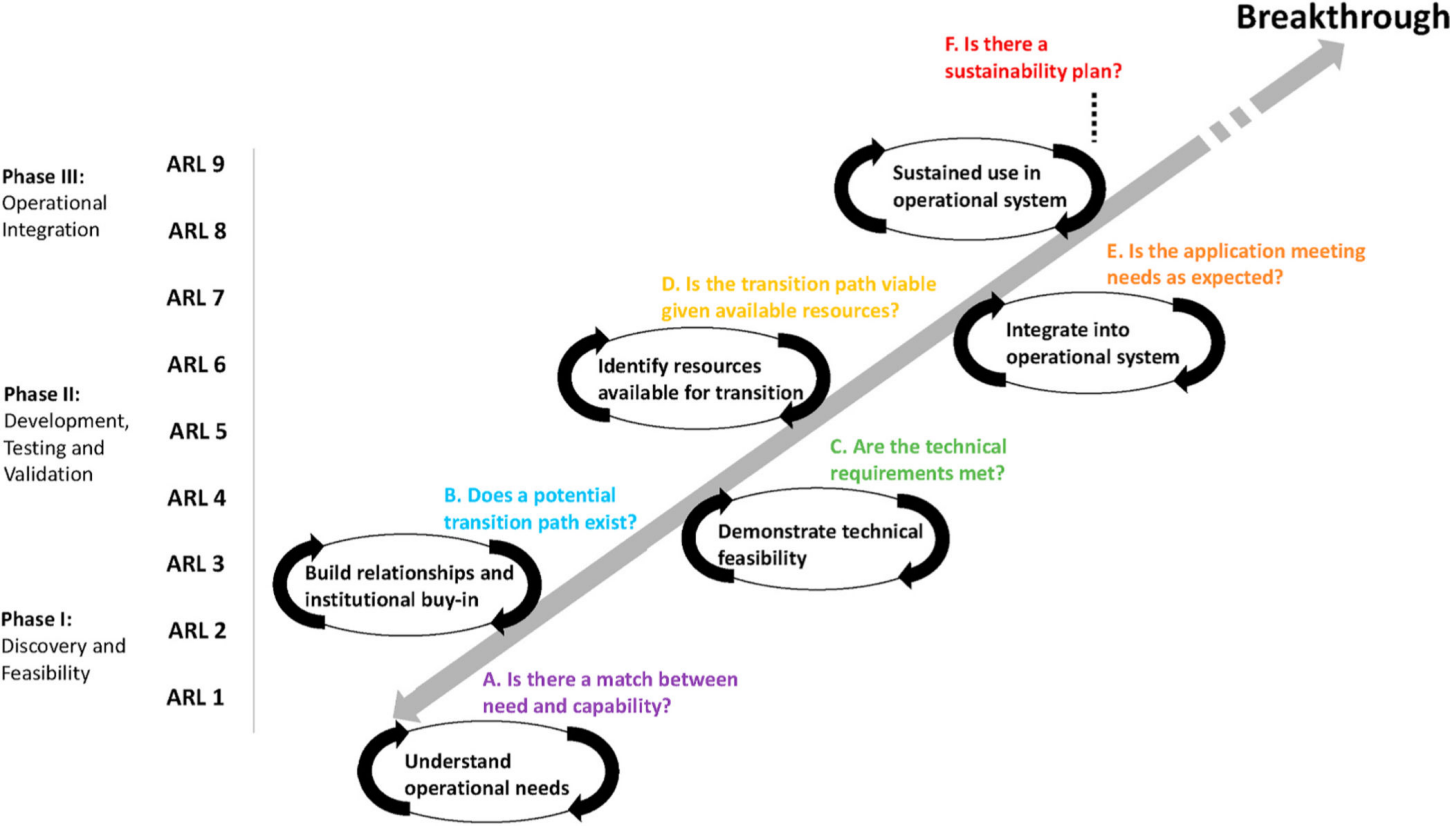
The NASA R2O–O2R framework, consisting of six iterative, linked processes that research and operational organizations co-develop. The framework builds on NASA’s ARL scale by folding in relationship-building requirements as well as technical milestones. Coupled together, these technical and social processes form the foundational elements needed to successfully transition Earth science from research to operations while also providing an explicit pathway for operational knowledge to inform research directions and practices.

Our R2O–O2R framework consists of six iterative, linked processes that are co-developed by the research and operational organizations involved (Fig. 3). An R2O breakthrough is achieved when each of the nine ARLs and six iterative processes are complete. En route through each one, both the research team and operational partner address multiple sets of questions, many of which are selected from the larger set of questions in the RIU framework [12]. Some of the questions correspond to issues unique to each partner while others reflect issues both partners address (Table 1). Although displayed sequentially and roughly mapped onto the ARL scale, most of the iterative processes and questions overlap at various points and for different periods of time. Furthermore, the iterative processes can be re-visited and moved between at any point along the R2O–O2R processes. In this way, the structure of our R2O–O2R framework modifies the stage-gate linear approach of the original NASA ARL scale.

The content and flexible ordering of the process outlined in Fig. 3 and Table 1 emerged from a synthesis of existing literature [12,64], interviews with researchers and operational partners involved in the two case studies in this paper, and discussions from break-out groups in a 2019 inter-federal-agency workshop on technology transfer for water management [14].

The R2O–O2R framework emphasizes the mutuality of individual relationships between researchers and decision makers. Each partner learns from the other in a highly dynamic, iterative collaboration, rather than through a unidirectional learning path from researcher to practitioner [64]. In Fig. 3, the gray two-sided arrow connecting the “Breakthrough” line back to “Understand operational needs” emphasizes the continual opportunity along the R2O trajectory to deepen understanding of operational needs, which may inform the existing effort and/or guide future applied research directions (in other words, advance O2R).

As championed in the RIU model, navigating diverging rationales of science (“discovering truth”) and environmental policy and management (“gaining power” or “solving a specific practical problem”) involves coming to terms with the fact that not all scientific results are relevant to environmental practitioners and not every policy or management demand may be solved by scientific research resources [12]. This warrants the need for extensive integration processes, represented here in process A: “Is there a match between need and capability?” CyAN, for instance, spent about nine months talking to its initial small group of collaborators to thoroughly understand their priorities and needs. While the ASO project was originally built using well-known data gaps, the details of how its information would be best distilled for operational use were developed through formal and informal communications over several years. Our conceptual framework incorporates this by identifying needs assessments as a critical first step in successful R2O. Only after understanding these needs does the research group assess technical capabilities as possible solutions (ARL 1–6).

The R2O–O2R framework’s approach of relationship-building and mutual learning reflects the way in which CyAN and ASO declared themselves to be a network of collaborators. The projects refrained from using the term “end-user”, understanding that it could engender an ‘us’ (scientist or researcher) versus ‘them’ (end-user) mentality. The term end-user also implies that the users are at the end of a process and that the product is fully developed and marketed, which is rarely the case in environmental research efforts.

Compelling matches between an operational need and a technical capability, as well as strong relationships between individuals, are necessary but not sufficient conditions for environmental KT/TT. Organizational and financial support is also necessary. The R2O–O2R framework emphasizes the simultaneous co-development of institutional support and financial planning alongside technical milestones (Process B: “Does a potential transition path exist?“). On the one hand, translating needs into technical requirements, developing accurate prototypes, simulating expected data, and conducting validation studies (ARL 3–5) help build trust and reinforce institutional support for the project to continue. On the other hand, the technical development, testing and initial integration of the science application (ARL 6–8; Process C) are only worth pursuing if the operational and research organizations have a strong working relationship and are able to reach a consensus that a viable transition path exists (Process B).

The operational partner primarily leads the process of translating the potential transition path into a verified and viable transition plan (Process D: “Is the transition path viable given available resources?“), though the research institution may also play a supporting role. This involves identifying financial, technical and human resources needed for transition.

Our framework expands the definition of what it means to undergo a successful transition from research to operations. NASA’s ARL system currently defines success (ARL 9) to occur when decision makers repeatedly use a new science capability or technological tool in their decision-making activities. However, repeated use does not guarantee that the application will be sustainable and available for operational use over the long-term. For this reason, the final phase of the R2O–O2R framework involves the formation of a plan detailing exactly how the scientific application would be made available throughout a specified timeframe [65]. This sustainability plan needs to consider the financial and technical resources that would be needed for long-term operational deployment along with appropriate ownership of the program. Though the R2O transition paths differed between CyAN and ASO, both took several years or more to develop and approve a plan for how the data/application would be provided in a sustained way for the duration of a future time frame. For ASO, the sustainability plan involved developing a revenue model and charting the institutional and legal steps to allow for the creation of Airborne Snow Observatories, Inc. For CyAN, agreements were established with NASA, NOAA and USGS to continue to deliver the required satellite products and support was secured internally within the EPA to continue hosting the web-based and mobile applications, and national cyanobacteria indicator metrics of temporal frequency [39,40], spatial extent [41], magnitude [42], and occurrence [43].

## 4. Discussion

Well-established university-to-industry pathways offer insights that can improve knowledge and technology transfer success rates among environmental researchers and decision makers. Our findings and the literature underscore the criticality of relational activities and the cultivation of trust on an individual level [14]. Existing work, including the “open-innovation hypothesis” and associated literature, highlight how collaborative external engagement – collaborative joint/contract research, consulting and informal interactions – often enables knowledge and technology transfer [10,66–69]. Moreover, the social relationships between individual organizational members usually catalyze organization-level relationships and associated institutional infrastructure in university-industry collaborations [70]. These findings are echoed by both ASO and CyAN. Our R2O–O2R framework recognizes this reality, filling existing gaps in the way NASA portrays KT/TT and pushing for an agency-wide culture that fosters collaborative relationship-building with individuals from outside organizations.

For long-term success, however, NASA and other environmental research and decision-making entities cannot solely rely on individual relationship-building or rare project champions [14]. Analysis of university-industry KT/TT [33] suggests that organization-level support is far more relevant for commercialization – licensing, patents and entrepreneurship – than collaborative external engagement, which tends to be driven by individuals and teams with little central support. We argue that this is not the case for environmental KT/TT. The two case studies presented here point to the importance of organizational support in both domains: commercialization *and* collaborative external engagement. Not only was the organizational support critical for ASO and CyAN, but the ASO project indicated that additional support would have improved KT/TT outcomes, particularly for accelerating utilization processes. While ASO greatly benefitted from the support provided by NASA’s Commercialization and Applied Sciences Programs, these institutional mechanisms did not provide guidance for some of the most complex and challenging aspects of KT/TT, including long-term planning, inter-institutional communication, and the establishment of a path towards revenue-generation in a context where traditional revenue models do not apply. CyAN benefited from organizational support, such as through well-established webinar series and communication channels. The project leveraged these established organizational communication channels across agencies [47]. Although ad-hoc approaches to navigating financial and communication challenges ultimately worked for ASO, systemic organizational support beyond the purview of existing KT/TT institutional mechanisms would have been greatly beneficial and arguably could lead to broader success as demonstrated in CyAN.

Expanding the NASA ARL scale into the R2O–O2R framework is a first step towards increasing institutional recognition that NASA scientists could benefit from additional intra- and inter-organizational support for integration and utilization activities. Such organizational support is particularly important in light of the unequal playing fields of gender and seniority that individual researchers must navigate [71–75]. NASA Applied Science solicitations require expert knowledge for activities such as stakeholder relationship-building, impact assessments, capacity building and transition planning. Few researchers receive formal training in these areas and are often overwhelmed by the many skills required to successfully complete applied research [14]. Each environmental research institute could develop their own version of training for KT/TT, but greater impact may come from national programs addressing this gap more systematically and efficiently. Such programs could provide formal training, peer-to-peer mentorship programs or expert consultations for applied researchers wanting to gain the skills needed to navigate the many barriers involved in environmental knowledge and technology transfer [64]. In addition to avoiding duplication of effort, environmental KT/TT training programs could lead to increased partnerships across and between environmental research institutions, as well as the cross-fertilization of ideas.

Our R2O–O2R framework also points to ways in which NASA Earth science could improve their career-incentive structures. To more accurately reflect the value of the work being done, career advancement could be evaluated beyond peer-review publication statistics to include indicators that directly reflect progress towards KT/TT [14,76]. These indicators could go beyond patents and licensing agreements to also reflect the relational processes that underpin successful non-commercialization knowledge transfer, including building social capital [77], memoranda of understanding, testimonials, letters of support for operationally incorporating a proposed new technology, and in-kind support/financial contributions from operational partners. Such measures are beyond the scope of NASA’s current ARL milestones. A NASA career incentive could resemble how government agencies typically handle contracts with performance reports: if the lead scientist achieves a higher ARL and/or associated relationship-building milestones, as outlined in our R2O–O2R framework, the reward could take the form of a weighted review on future proposals. One important caveat would be to prevent stove piping with only a few investigators being successful. Another route could involve the provision of grant amendments for publication-writing to supplement salaries. This could help offset the time spent on the relationship-building aspects of projects so that researchers can focus on career advancement and learning.

A notion pervasive in many research institutions is that KT/TT activities reduce the academic productivity of the scientists involved. However, this is often not the case. Most studies report that faculty with industry partnerships publish at least as many scientific articles as their colleagues, sometimes more [78,79]. Collaborative projects also often yield valuable outcomes that do not directly result in publishable results [80,81]. Rather than avoiding many forms of collaborative engagement, NASA individuals and programs that focused on basic research could instead consider establishing research partnerships with agreements designed to foster open-ended research [10]. In other words, collaborative KT/TT efforts can be structured to meet basic research objectives associated with gaining new knowledge for the sake of the knowledge itself instead of pursuing a specific technical, social or economic purpose [82].

Our work offers a conceptual advance in the field of environmental KT/TT, and charts a path for improving KT/TT outcomes for NASA. The best practices we highlight are qualitatively supported by the two case studies discussed above. An additional applied, qualitative measure illustrating the utility of the R2O–O2R framework is that many (~20–50) NASA environmental projects are already applying these best practices to successfully deliver KT and/or TT (examples below). Opportunities to quantitatively prove the efficacy of our framework will emerge as the number of NASA efforts adopting these principles continues to increase.

Examples of NASA environmental projects and partnerships currently adopting R2O–O2R best practices include:

### Navajo Nation Drought Tool –

NASA has developed a Drought Severity Evaluation Tool (DSET) with the Navajo Nation, which helps the community monitor drought and allocate emergency relief when drought hits. DSET is currently being used by the Navajo Nation’s Department of Water Resources in the face of ongoing extreme drought in the western U.S. and drought emergency declarations. The online tool uses precipitation data from NASA satellites, drought indices and ground-based rain measurements.

### SPoRT Project –

NASA is working with a host of U.S. government agencies, including the U.S. National Weather Service (NWS), to transition over 40 NASA satellite datasets and research capabilities to operational weather entities. The ultimate goal is to improve short-term operational weather forecasting and decision making to benefit society. SPoRT adopts the R2O–O2R principles outlined in this paper by focusing on end-user involvement (advocates, training, product assessments, user feedback) and by developing testbed environments in which operations can shape future research. Over 30 U S. NWS offices and multiple national NWS centers are involved.

### Crop-CASMA Soil Data Portal –

Working with the U.S. Department of Agriculture (USDA), NASA has launched a tool called Crop Condition and Soil Moisture Analytics System (Crop-CASMA, [83]). This web GIS tool provides high-resolution, field-scale soil wetness in an easy-to-use format, based on data from NASA’s Soil Moisture Active and Passive satellite mission. Crop-CASMA is helping the USDA move from using weekly surveys that offer qualitative soil moisture at county scale to quantitative estimates at high-resolution scale (1-km, 2–3 days). The new data augment soil moisture information in the USDA’s Crop Weather Report, which is based on subjective and qualitative field observations that rely on volunteer respondents and do not provide geospatial coverage information.

### Carbon Mapper –

This non-governmental non-profit with origins tied to NASA research enables mitigation of greenhouse-gas emissions by identifying, quantifying and monitoring global methane and carbon-dioxide (CO_2_) point-source emissions on the scale of individual facilities and equipment. To do this, Carbon Mapper is working to deploy a constellation of satellites and aircraft equipped with high-performance visible/infrared imaging spectrometers in partnership with NASA JPL as well as other partners from the private, nonprofit and philanthropic sectors. The resulting information allows for improved mitigation of methane and CO_2_ emissions and the potential delivery of many other hyperspectral indicators including support for ecosystem management.

### NASA Earth Science “Space For U.S.” –

NASA published “Space For U.S.”, a website that features 56 stories across the United States about how people are finding solutions to local challenges using NASA Earth observations [86]. The stories on the “Space for U.S.” provide numerous examples of NASA activities that are already promoting many aspects of our R2O–O2R framework as a recipe for successful use of remote-sensing data to advance environmental knowledge and technology transfer.

## 5. Conclusion

In today’s rapidly-changing world, many communities are under increasing pressure to help better manage our natural resources. Here, we offer a conceptual advance in the field of environmental KT/TT, and chart a path for improving KT/TT outcomes for NASA, a key U.S. agency working in the environmental realm. We qualitatively support our framework with two case studies as well as a number of on-going NASA KT/TT projects that are already applying the R2O–O2R framework best practices. As the sample size of such projects increases, it will be possible for future studies to deliver a rigorous, quantitative validation of the R2O–O2R framework.

The two case studies presented show that insights gained from university-industry KT/TT can help research transfer to environmental organizations more effectively, delivering solutions to those on the ground who are making natural-resource stewardship choices. In recognition of these insights, environmental researchers and decision-making agencies or organizations can create an organizational culture that fosters long-term relationship building and collaborative, two-way knowledge exchange with individuals from outside organizations.

Traditional KT/TT pathways are framed mainly within a commercial or industry context. Many environmental organizations are trying to do KT/TT for common-pool resources that may not involve profit-oriented factors or viable commercial ventures, but are instead facilitated by intervention primarily from governments or non-profits. While some work exists on the topic of successful environmental KT/TT untied from commercial outcomes [11,84,85], more research is needed [14,17]. Our findings add to the limited body of knowledge on this topic, showing how the goals, resources and opportunities of environmental KT/TT can differ from more traditional university-private sector pathways.

To navigate the dynamic tension between well-established KT/TT pathways and the unique issues environmental organizations face, we propose a strategy for incorporating KT/TT best practices at the organizational level – in a way that builds on existing institutional strengths, culture and history. Our R2O–O2R framework illustrates what this might look like for NASA and provides recommendations for improving how the agency harnesses its satellite and climate expertise to promote environmental KT/TT. Other environmental research and operational entities can adopt a similar strategy of building on existing institutional logic to advance KT/TT outcomes.

## Supplementary Material

Supplement1

## Figures and Tables

**Table 1:** Six sets of questions (A-F) correspond to the six iterative processes shown in Fig. 1. The questions help ensure a successful partnership between research and operational teams, with shared agreement about how the collaboration can advance toward sustained operational use. Each overarching question (column 1) corresponds to a subset of questions that the research and operational partners address together (column 2) and individually (columns 3–4). These relationship requirements underpin advances in the ARL of the project.

Breakthrough	Both partners	Operational partner	Research partner
**A. Is there a match between need and capability?**	Is there interest from both partners to further explore meeting the identified needs?	What are our highest priority needs? Can the research partner communicate clearly and effectively about their capabilities?	Can the operational partner communicate clearly about their needs? Are there any capabilities that might meet operational needs and requirements?
**B. Does a potential transition path exist?**	Is there a strong personal connection between research and operational teams? Is there a sense of mutual respect and commitment to joint success? Is there enough evidence of institutional support from both parties to proceed?	Does the research partner accept input along the way? Will the research partner be willing to spend one on one time with us? Is the research partner sincerely interested in helping our program or will the scientist “publish, present and perish”?	Does partner show sufficient interest and commitment? Does this project sufficiently advance career?
**C. Are the technical requirements met?**	Will the accuracy, resolution and associated uncertainty of the proposed product or technology meet operational needs?	Does science team openly discuss data limitations and issues? Can we test and validate data ourselves? Can we meet in person with the science team and other users to work through issues?	Can operational needs be translated into technical requirements? What is the best approach to validate these requirements?
**D. Is the transition path viable given available resources?**	What are the financial, technical and human resources needed for a successful transition? Is there sufficient institutional buy-in to proceed?	Are there compelling high-level summaries, impact assessments as well as technical details available to share with upper management? Do we have the financial, technical and human resources needed?	Are there any ways the research organization can help secure support for transition within or outside the operational partner organization?
**E. Is the application meeting needs as expected?**	Is the accuracy, resolution and uncertainty of the product at the expected levels? What capacity building is needed?	What additional refinements are needed? Does science team continue to take initiative to talk to collaborator?	Can the identified refinements be delivered given the timing and scope of project?
**F. Is there a sustainability plan?**	Is it feasible given available resources as well as known risks and uncertainties?	Is additional funding needed to enact the sustainability plan? How can the funding of this application be leveraged with other internal programs?	Are there any additional capacity building efforts needed to ensure success of the sustainability plan?
