# Supplementary material for: Paths to research-driven decision making in the realms of environment and water
Source: Technol Soc. Author manuscript; Available in PMC 2024 Jul 17. (PMC11252905; doi:10.1016/j.techsoc.2022.101994)
Supplement: Supplement1 [file NIHMS1877250-supplement-Supplement1.docx]

# **Supplemental Information**

**Figure S1**. NASA Applied Sciences Program Application Readiness Level (ARL) scale (Friedl, 2018). The progression of projects is represented vertically (y-axis). While some projects may reach milestones out of phase with this upwards vertical progression, the ARL for each project is determined at any given time by the completion of all milestones that come below it in this illustration.


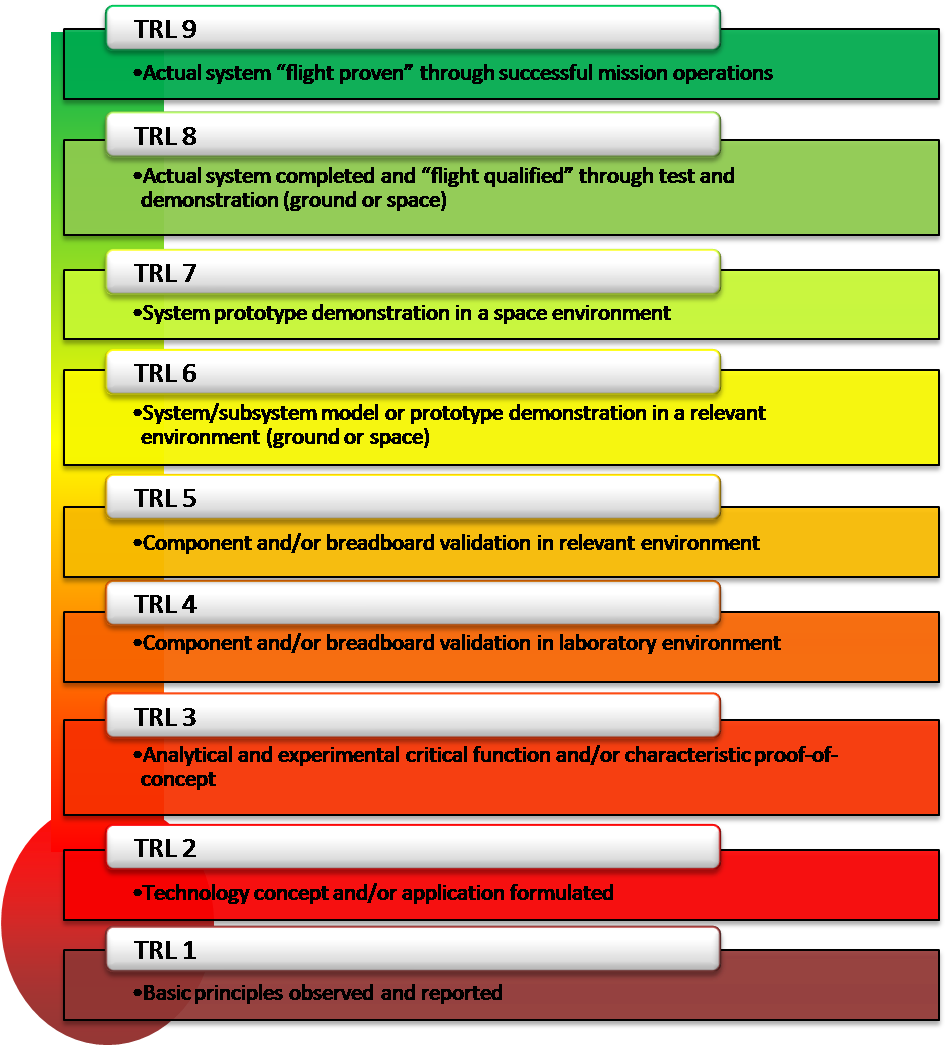


**Figure S2**. NASA’s nine Technical Readiness Levels (TRLs) are used to assess the maturity level of a particular technology (Mankins, 1995; Frerking & Beauchamp, 2016). The TRL scale ranges from 1 – where scientific research is beginning and the results are being translated into future research and development – to 9 – at which point a technology has been "flight-proven" during a successful mission.
